# Selective laser melting fabrication of functionally graded macro-porous Ti-6Al-4V scaffold for cavity bone defect reconstruction

**DOI:** 10.3389/fbioe.2025.1550309

**Published:** 2025-04-28

**Authors:** Zhuangzhuang Li, Yi Luo, Ruicheng Liu, Shanfang Zou, Yitian Wang, Taojun Gong, Xuanhong He, Yong Zhou, Minxun Lu, Li Min, Chongqi Tu

**Affiliations:** ^1^ Department of Orthopedics, Orthopedic Research Institute, West China Hospital, Sichuan University, Chengdu, Sichuan, China; ^2^ Model Worker and Craftsman Talent Innovation Workshop of Sichuan Province, Chengdu, Sichuan, China; ^3^ Tianqi Additive Manufacturing Co., Ltd., Chengdu, Sichuan, China

**Keywords:** Ti-6Al-4V, functional graded porous, cavitary bone defect, autologous cancellous bone matrix, selective laser melting

## Abstract

Reconstruction of cavitary bone defects poses significant challenges in orthopedic surgery due to the irregular shapes and compromised mechanical properties of surrounding bone. This study developed a functionally graded macro-porous scaffold (FGMPS) using selective laser melting (SLM) for cavitary bone defect reconstruction. The FGMPS featured a porosity gradient (74%–86%) and macropores ≥1,600 µm, mimicking the natural density gradient of cancellous bone. Micro-CT analysis confirmed high structural fidelity and interconnected porosity. Compression tests in two orientations revealed distinct stress-strain responses: vertically aligned gradients (FGMPS-V) exhibited sequential layer engagement, while horizontally aligned gradients (FGMPS-H) demonstrated higher stiffness and strength due to uniform load distribution. The elastic modulus ranged from 383 MPa (FGMPS-V) to 577 MPa (FGMPS-H), with yield strength of 22–40 MPa, aligning well with cancellous bone properties. These findings highlight the FGMPS’s potential to offer a promising solution for cavitary bone defect repair.

## 1 Introduction

Reconstruction of critical-sized bone defects remains a significant challenge in orthopedic surgery, often necessitated by tumor resection, trauma, or congenital anomalies (Dalfino et al., 2023; Tarchala et al., 2016; Wang and yeung, 2017). Specifically, based on morphology, critical-sized bone defects can be categorized into segmental bone defects and cavitary bone defects (Huang et al., 2022). Segmental bone defects are characterized by the complete loss of a section of the bone, typically disrupting the bone’s load-bearing capacity. Reconstruction of such bone defects often involves the use of segmental autogenous/allogeneic bone grafts or metallic implants to restore both the mechanical integrity and biological function of the affected bone (Mauffrey et al., 2015; Zhang et al., 2020; Li et al., 2024a). In contrast, cavitary bone defects are hollow spaces within the metaphyseal that result from conditions such as cysts or the curettage of benign tumors like giant cell tumors of bone (GCTB) and osteoblastoma (Horstmann et al., 2018). These defects are often more localized but pose unique challenges due to their irregular shapes and the surrounding bone’s compromised mechanical properties. Cavitary defects require not only filling but also integration with the surrounding bone, while maintaining the mechanical stability necessary for load-bearing regions (Zhang et al., 2021). Traditional treatment methods often involve the use of autogenous cancellous bone graft or cement filling; however, these options are associated with several limitations. Autogenous cancellous bone graft, while being the gold standard, often fail to provide the necessary structural support and is limited by limited availability (Robinson et al., 2020). Cement filling, on the other hand, may suffer from mismatched elastic modulus with metaphyseal cancellous bone, leading to eventual loosening or failure (López et al., 2014).

In recent years, the use of biomaterials and tissue engineering approaches has gained attention as promising alternatives for bone defect reconstruction. Among the various biomaterials investigated, titanium alloy (Ti-6Al-4V) has emerged as materials of choice in orthopedic applications due to the excellent mechanical properties, biocompatibility, and corrosion resistance (Kaur and Singh, 2019; Aufa et al., 2022; Hoque et al., 2022; Zhang X. et al., 2018; Trevisan et al., 2018). However, ensuring optimal integration of Ti-6Al-4V implants with host bone and promoting osteogenesis remains a significant challenge. One of the key factors influencing implant success is the design of the porous architecture (Chung and King, 2011; Wang et al., 2016). Many types of porous structures have been investigated, such as body-centered cubic (BCC), face-centered cubic (FCC), triply periodic minimal surfaces (TPMS), and irregular Voronoi-based structures (Chen et al., 2020; Lv et al., 2021; Chao et al., 2023). These designs have been shown to achieve mechanical adaptability, with suitable elastic modulus and satisfactory strength. It is noting that natural bone is not a homogeneous structure; it exhibits a gradient in both density and mechanical properties (Currey, 2012; Breish et al., 2024). Recently, the functional graded porous scaffold (FGPS) has been proposed, which could mimic the natural gradient of bone structure (Wang et al., 2016). Many studies have demonstrated that FGPS offer significant advantages over uniform porous scaffold (UPS) in terms of mechanical performance. For example, mechanical testing has demonstrated that FGPS exhibit superior fatigue performance, which makes them more durable under cyclic loading for long-term orthopedic applications (Chen et al., 2023). Additionally, in the design of novel orthopedic implant, FGPS could effectively distribute mechanical loads and mitigate stress concentrations at the bone-implant interface, thus enhancing adaptation to physiological load requirements and reducing the risk of stress shielding and bone resorption (Naghavi et al., 2023).

At present, FGPS primarily designed for segmental bone defects has been investigate thoroughly, which overlapped the mechanical properties of both cortical and cancellous bone (Zhang X-Y. et al., 2018). However, cavitary bone defects primarily affect cancellous bone in the metaphyseal region, with most surrounding cortical bone remaining intact. This necessitates scaffolds with specific mechanical properties tailored to the unique demands of metaphyseal bone. Reducing the elastic modulus of such Ti-6Al-4V scaffolds to match cancellous bone can be achieved by decreasing strut diameter or increasing pore size to achieve higher porosity. While decreasing strut diameter may reach the limits of additive manufacturing and increases the risk of printing failure (Caiazzo et al., 2022), increasing pore size offers a more feasible alternative. High-porosity scaffolds with larger macro-pores not only effectively reduce overall stiffness to better match the elastic modulus of cancellous bone but also provide space to carry autologous cancellous bone matrix. This matrix, rich in cytokines and osteoinductive components (Georgeanu et al., 2023), supports biological integration while minimizing risks of immune rejection and disease transmission. By combining functionally graded macro-porous scaffolds (FGMPS) with autologous cancellous bone matrix, this approach holds great promise for addressing both the structural and biological requirements of cavitary bone defect reconstruction in a safe and personalized manner.

In this study, we aimed to develop and evaluate a FGMPS based on a dodecahedral unit cell design. The FGMPS was designed greater than 1,600 µm macropores and fabricated using selective laser melting (SLM). Comprehensive microstructural and mechanical analyses were conducted to assess its suitability for reconstruction of cavitary bone defects. Our findings demonstrate the potential of this scaffold as an advanced, bioactive solution for cavitary bone defect reconstruction in orthopedic surgery.

## 2 Methods

### 2.1 Graded porous structure design scheme

**FIGURE 1:**
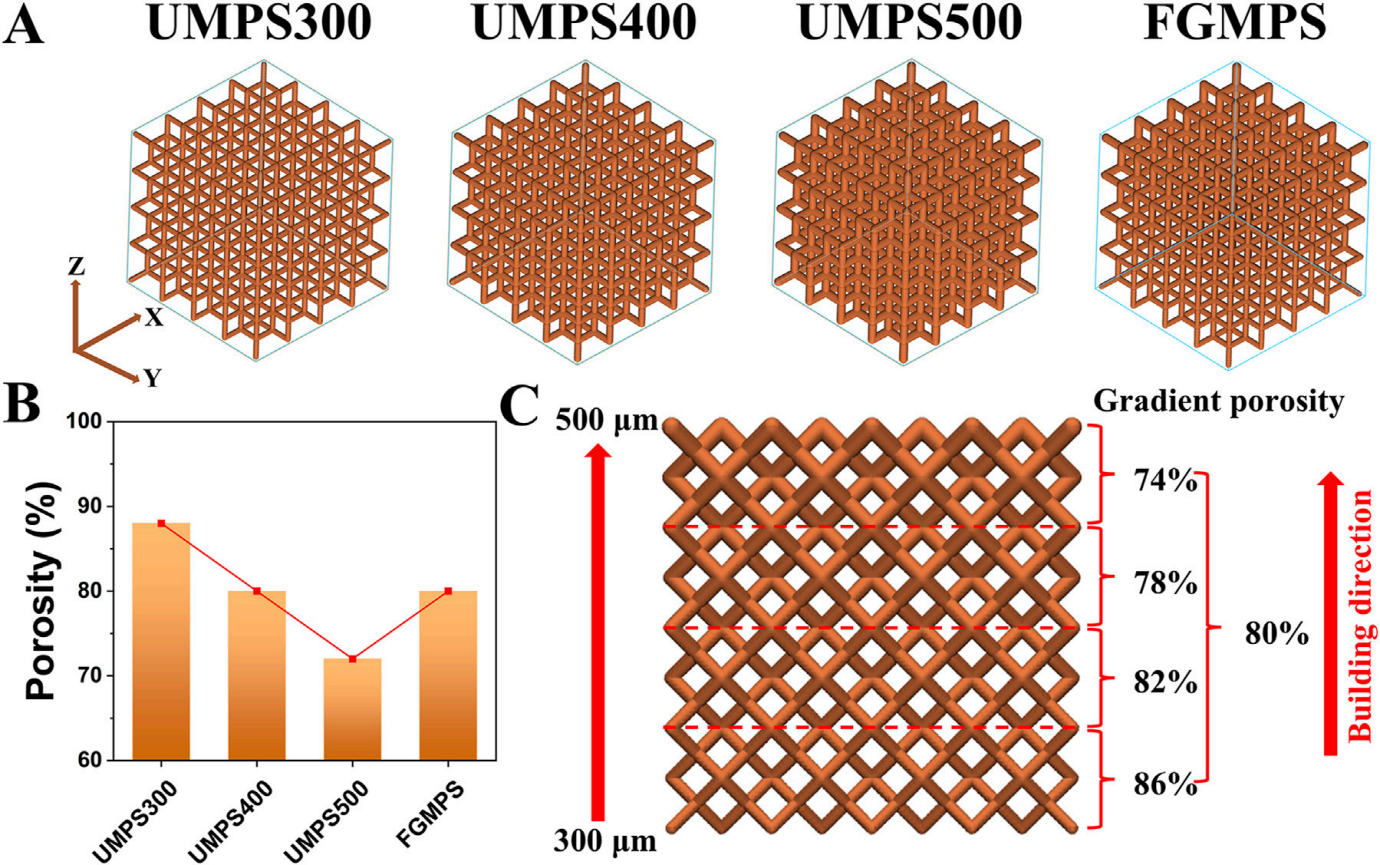
Design and porosity analysis of uniform and functionally graded macro-porous scaffolds. **(A)** 3D models of the four scaffolds, including uniform macro-porous scaffolds with strut diameters of 300 μm (UMPS300), 400 μm (UMPS400), and 500 μm (UMPS500), as well as a functionally graded macro-porous scaffold (FGMPS) with a gradient in strut diameter. **(B)** Porosity comparison of the UMPS300, UMPS400, UMPS500, and FGMPS scaffolds, showing porosities of 88%, 80%, 72%, and 80%. **(C)** Illustration of the FGMPS scaffold’s layered porosity distribution, with values of 74%, 78%, 80%, 82%, and 86% along the vertical building direction.

In this study, four distinct porous scaffolds—uniform macro-porous scaffold-300 (UMPS300), UMPS400, UMPS500, and functionally graded macro-porous scaffold (FGMPS)—were designed based on the dodecahedral unit cell, which is commonly used in orthopedic porous implants (Li et al., 2024b; Hou et al., 2020), as shown in Figure 1A. The dodecahedral unit cell was selected for its superior biomechanical properties compared to BCC structures, as its high connectivity and uniform stress distribution help reduce localized stress concentrations. Additionally, compared to TPMS, which offer excellent mechanical properties but require complex manufacturing controls, the dodecahedral unit cell provides greater predictability and reproducibility in large-scale fabrication using selective laser melting SLM. These advantages make it a promising choice for achieving biomechanical compatibility with cancellous bone.

Each scaffold was composed of repeating dodecahedron unit cells, with the overall dimensions of 10 × 10 × 10 mm. The key design variable across these scaffolds was the strut diameter of the dodecahedral cells. For the UMPS300, UMPS400, and UMPS500, the strut diameters were set to 300 μm, 400 μm, and 500 μm, respectively. The FGMPS, on the other hand, was designed with a gradient in strut diameter, creating a porosity profile that changes gradually across its structure. As illustrated in Figure 1B, the porosities of UMPS300, UMPS400, and UMPS500 were approximately 85%, 75%, and 70%, while the FGMPS scaffold exhibited a layered gradient porosity of 74%, 78%, 80%, 82%, and 86% along the vertical build direction, as shown in Figure 1C. The pore size was assessed using the ‘pore sphere’ method within the unit cell of the lattice structure (Supplementary Material S1). The pore size of each scaffold was summarized in Table 1.

**TABLE 1:** Pore sizes of designed UMPS300, UMPS400, UMPS500, and FGMPS scaffolds.

Scaffold type	Pore size (µm)
UMPS300	1800
UMPS400	1700
UMPS500	1,600
FGMPS	1,660–1780

### 2.2 Powder materials and SLM process

**FIGURE 2:**
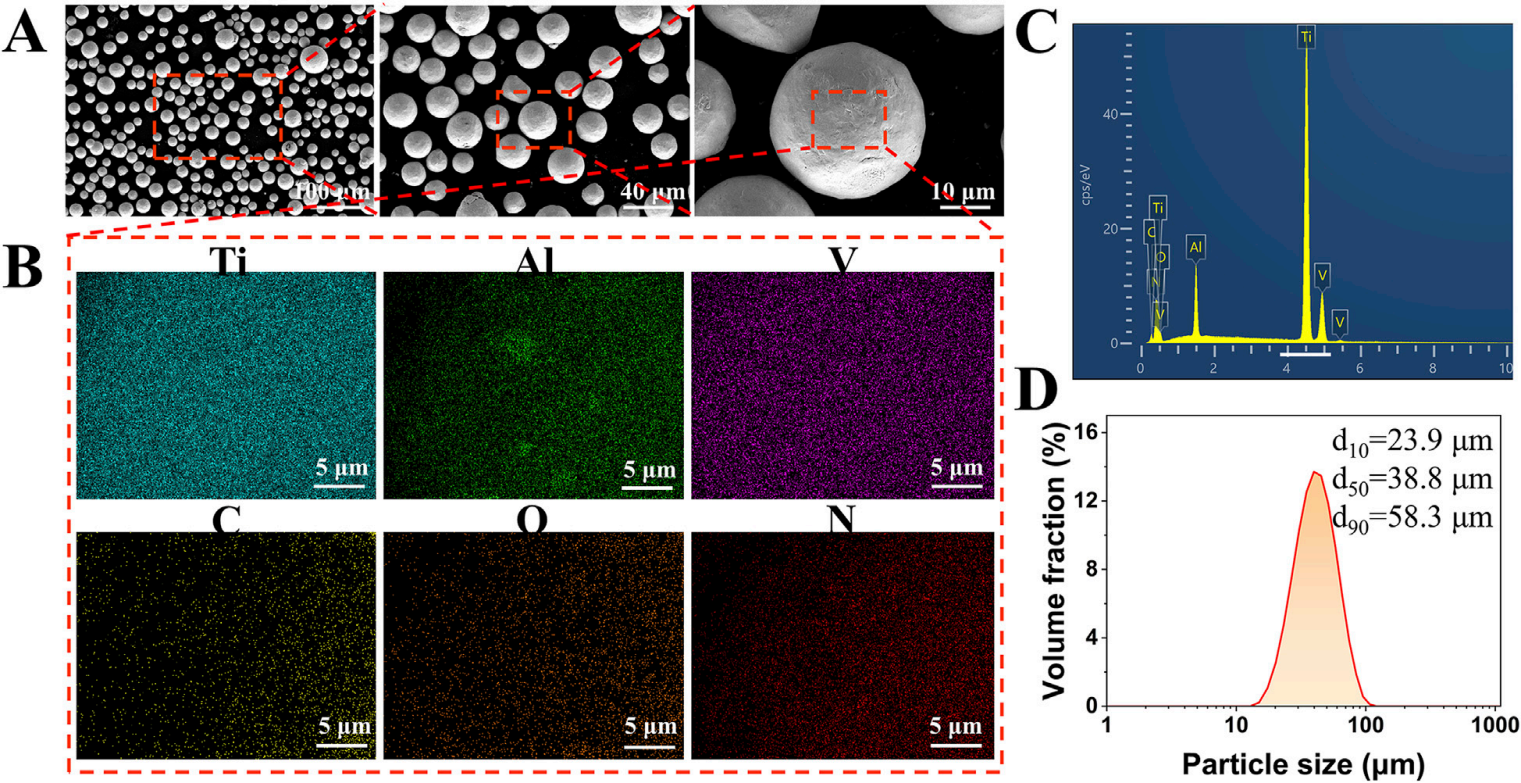
Characterization of Ti-6Al-4V alloy powder used for scaffold fabrication. **(A)** SEM images showing the predominantly spherical morphology of the Ti-6Al-4V powder particles. **(B)** Elemental mapping confirms the homogenous distribution of Ti, Al, and V, with trace amounts of C, O, and N. **(C)** EDS spectrum of the Ti-6Al-4V powder shows prominent peaks of Ti, Al, and V, indicating the primary alloy composition. **(D)** Particle size distribution of the powder determined by laser diffraction analysis, with values of d_10_ = 23.9 µm, d_50_ = 38.8 µm, and d_90_ = 58.3 µm.

The raw powder material used for the scaffold fabrication was Ti-6Al-4V alloy powder, which was produced using electrode induction-melting gas atomization process. The scanning electron microscopy (SEM) images in Figure 2A showed that the powder particles were predominantly spherical. Detailed elemental mapping analysis confirmed a uniform distribution of Ti, Al, and V, with minor amounts of C, O, and N (Figure 2B). The chemical composition of the Ti-6Al-4V powder was analyzed by energy-dispersive X-ray spectroscopy (EDS), as shown in Figure 2C. The EDS spectrum revealed prominent peaks of Ti, Al, and V, confirming the primary constituents of the alloy powder. The particle size distribution of the Ti-6Al-4V powder was determined using laser diffraction analysis, as presented in Figure 2D. The results indicated that the powder had a d_10_ of 23.9 µm, a d_50_ of 38.8 µm, and a d_90_ of 58.3 µm. The particle size range was well-suited for the SLM process, facilitating consistent powder layer deposition, which is vital for achieving high-quality and reliable performance in the final scaffold structure (Murr et al., 2012). The Ti-6Al-4V scaffolds were fabricated using a M1 machine (Concept Laser, Germany), under a high-purity argon atmosphere (purity >99.99%). The employed parameters were summarized in Table 2.

**TABLE 2:** SLM process parameters employed in this work.

Processing parameters	Values
Laser power	190 W
Scanning speed	1,200 mm/s
Layer thickness	30 μm
Hatching space	100 μm
Spot diameter	110 μm

### 2.3 Characterization of scaffold morphology

After fabrication, all samples underwent sandblasting to remove un-melted particles on the surface. Then, the scaffolds underwent ultrasonic cleaning and were thoroughly dried. The actual porosity of the samples was determined using the dry weighing method. An analytical balance was used to measure the mass of each specimen, and the porosity of the tested scaffold was calculated using Equation 1 (Melancon et al., 2017):

$$\mathrm{Porosity}=\left(1-\frac{m_{s}}{\rho_{t}\times V_{d}}\right)$$ (1)

where *m*_*s*_ is the mass of each sample, *ρ*_*t*_ represents the theoretical density of Ti-6Al-4V (4.42 g/cm^3^), and *V*_*d*_ is the volume of the design domain (1 cm^3^). The experiment was repeated in triplicate (n = 3) and presented as mean ± SD.

To further characterize scaffold morphology, SEM was employed using the Zeiss EVO 10 SEM (Germany). The surface morphology and microstructural features of the scaffolds were observed.

### 2.4 Micro-CT analysis

The internal architecture of the scaffolds was analyzed using a GE Nanotom micro-computed tomography (Micro-CT) system with a tube voltage of 140 kV, current of 100 µA, and exposure time of 1,000 ms, achieving a voxel size of 5 µm. The 3D models were reconstructed in VGStudio MAX 3.1 to evaluate strut distribution, pore interconnectivity. Surface deviation analysis was conducted by comparing the reconstructed models with the original CAD designs, generating 3D color maps. The average deviation values (D_50_) were calculated to assess printing accuracy for each scaffold type.

To investigate the internal micro-pore defects, the segmented Micro-CT data were used to quantify pore morphology, volume, and distribution. The pore volume and its relationship with sphericity were analyzed across different scaffold types.

### 2.5 Compression test

The compressive mechanical properties of the porous scaffolds were evaluated through uniaxial compression tests. Specifically, the FGMPS were tested under compression in two distinct orientations: one where the gradient direction was aligned with the compression axis (gradient vertical direction; indicated as FGMPS-V), and another where the gradient direction was perpendicular to the compression axis (gradient horizontal direction; indicated as FGMPS-H). All tests were performed at a compression speed of 0.2 mm/min using an Instron 5982 universal testing machine (Instron, USA). The experiment was repeated in triplicate (n = 3). The engineering strain (*ε*) of the specimen was calculated using Equation 2:

$$\varepsilon =\frac{\Delta L}{L_{0}}$$ (2)

where *ε* represents the engineering strain, *ΔL* is the change in length of the specimen, and *L*_*0*_ is the original length of the specimen (10 mm). The elastic modulus was calculated as the slope of the linear portion of the stress-strain curve, while the yield strength was determined using the 0.2% offset method. The ultimate strength was defined as the first peak stress achieved before failure.

### 2.6 Statistical analysis

Statistical analyses were conducted utilizing SPSS statistics software (version 22; IBM Corp., Armonk, NY, USA). The results were presented as mean ± deviations.

## 3 Results and discussion

### 3.1 Morphology characterization

**FIGURE 3:**
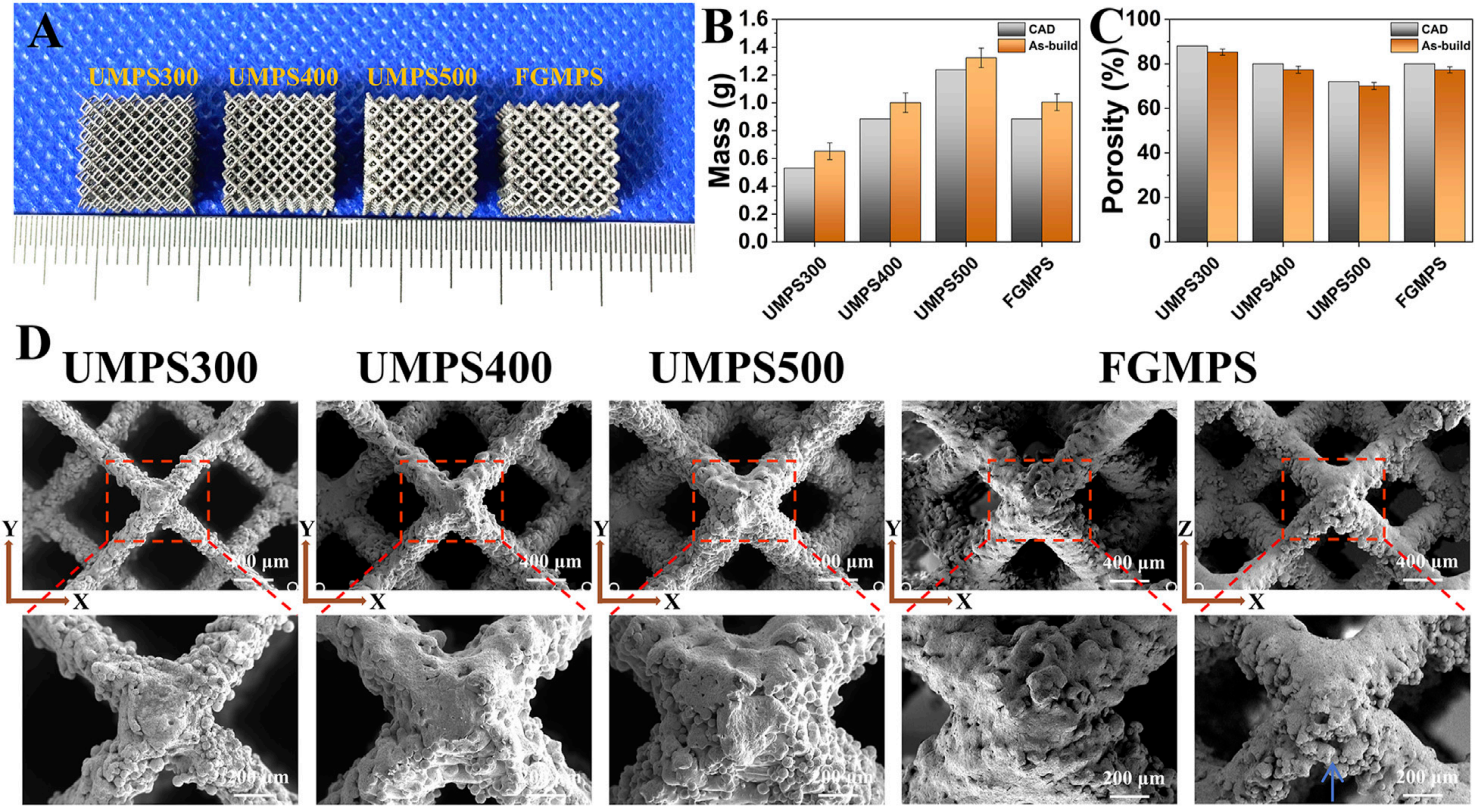
**(A)** Images of as-built samples (UMPS300, UMPS400, UMPS500, and FGMPS). **(B)** Mass measurements reveal that as-built samples have consistently greater mass than CAD models. **(C)** Porosity analysis indicates that as-built samples have lower porosity compared to CAD designs. **(D)** SEM images of scaffold show dodecahedral unit cells with different strut diameters (UMPS300 to UMPS500) and the gradient structure of FGMPS.

Figure 3 shows that the as-built samples demonstrated differences in both mass and porosity compared to the CAD designs. Specifically, the as-built samples consistently exhibit greater mass and reduced porosity across all groups (UMPS300 to FGMP). This indicates that the actual structures are denser and less porous than originally designed. Our findings align with the study of Zhang X-Y. et al., (2018), which also reported increased mass and decreased porosity in the as-built samples compared to CAD designs. This may be attributed to particle adherence on the struts during SLM process.

The SEM images (Figure 3D) provide detailed insights into the surface topology, strut connections, and pore architecture of the scaffolds. The images clearly illustrate the dodecahedral unit cell arrangement and highlight the effect of increasing strut diameters from UMPS300 to UMPS500, resulting in a corresponding decrease in pore sizes. The lateral morphology of the GMPS scaffold demonstrates the gradual transition of strut diameter from one layer to the next, contributing to its graded structure. Notably, a significant distribution of adhered powder can be observed around the struts, primarily accumulating along their periphery. The primary factor is the thermal effect during SLM. The high temperatures generated by the laser can cause nearby powder particles to partially melt and adhere to the scaffold struts (Debroy et al., 2018). In the lateral view, it is observed that the adhered powder tends to accumulate primarily along the lower regions of the struts (as indicated by the blue arrow). This phenomenon could be contributed to the gravitational effects during the powder bed fusion process, and powder particles naturally settle along the lower regions under gravity (Yap et al., 2015; Spierings et al., 2013).

### 3.2 Surface deviation

**FIGURE 4:**
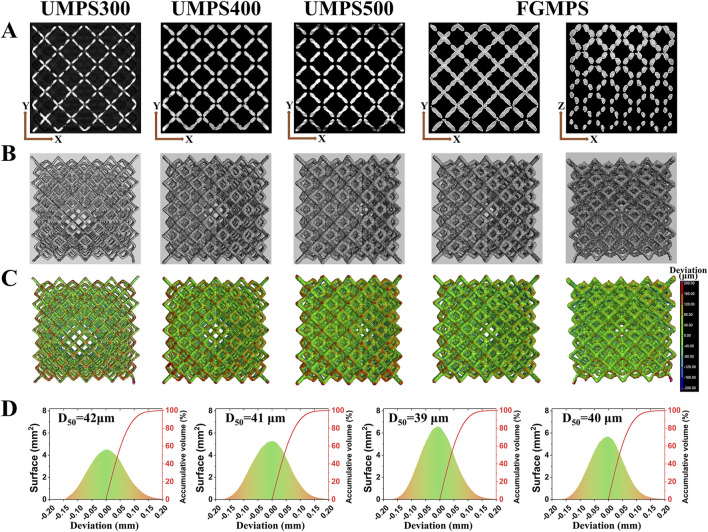
Micro-CT analysis and surface deviation of scaffolds. **(A)** Micro-CT images showing the internal structure of each scaffold (UMPS300, UMPS400, UMPS500, and FGMPS), with uniform strut distribution and interconnected pores. **(B)** The FGMPS scaffold shows a gradient in strut diameter, creating a graded porosity from base to top. **(C)** 3D color maps of surface deviations compare the fabricated scaffolds to CAD designs. **(D)** Average deviation values (D50) for UMPS300, UMPS400, UMPS500, and FGMPS are 42 μm, 41 μm, 39 μm, and 40 μm, respectively.

Micro-CT images (Figure 4A) provide a comprehensive visualization of the internal architecture of each scaffold, highlighting the uniformity in strut distribution and the interconnected nature of the pores. The gradient porosity of the FGMPS scaffold is particularly evident, characterized by a gradual variation in strut diameter from the base to the top. This progressive change in strut diameter forms the graded architecture, aiming to replicate the natural density gradient and enhance biomechanical compatibility. While a direct comparison of pore interconnectivity between the FGMPS scaffold and natural cancellous bone is difficult due to the inherent complexity of cancellous bone structure, micro-CT analysis has confirmed that the FGMPS scaffold possesses an interconnected pore network. The 3D reconstruction of the scaffolds (Figure 4B) clearly demonstrates the continuous connectivity of pores, essential for facilitating bone in-growth and nutrient exchange.

Figure 4C presents 3D color maps generated using VGStudio software, which illustrate surface deviations from the designed geometry. These deviation maps reveal that the fabricated scaffolds closely matched their original CAD designs, with minimal surface discrepancies. The average deviation values (D50) for UMPS300, UMPS400, UMPS500, and FGMPS are determined to be 42 µm, 41 µm, 39 µm, and 40 µm, respectively, as depicted in Figure 4D. The deviations fall within acceptable limits for biomedical applications, demonstrating the precision of the SLM process, which is consistent with the precision levels reported by Li et al. for additively manufactured Ti-6Al-4V scaffolds (Li et al., 2023).

### 3.3 Micro-pore defect

**FIGURE 5:**
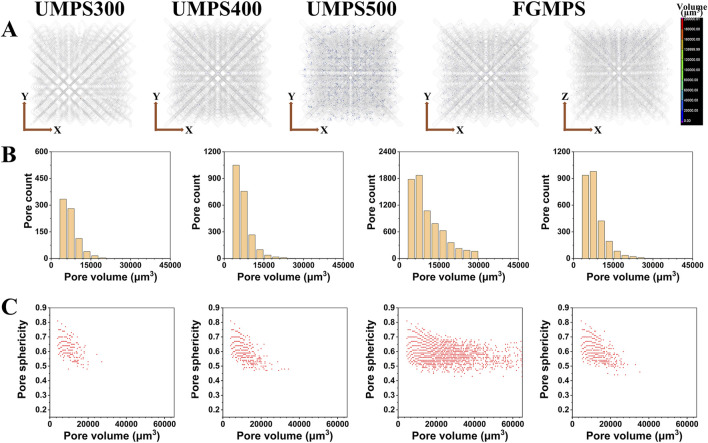
Micro-pore defect analysis of SLM-fabricated scaffolds. **(A)** Internal micro-CT images showing randomly distributed micro-pore defects within the struts of each scaffold type (UMPS300, UMPS400, UMPS500, and FGMPS). **(B)** Pore volume distribution for each scaffold present the count of pore defect. **(C)** Relationship between pore volume and sphericity, where the overall sphericity remains high.

To investigate the quality of scaffolds fabricated using the SLM process, this study conducted a detailed analysis of the internal structure of the struts to assess the characteristics and distribution of micro-pore defect and its potential impact on scaffold performance. As illustrated in Figure 5A, the internal micro-pore defects appear randomly distributed within the struts. These micro-pore defects are a common occurrence in the SLM process, primarily resulting from gas entrapment within the melt pool, which originates either from the powder material or forms during the rapid solidification process (Gao et al., 2022; Yeganeh et al., 2023). Therefore, random dispersion of spherical pores is observed throughout the structure. The pore volume distribution, as depicted in Figure 5B, highlights that scaffold with larger strut diameters (e.g., UMPS500) generally exhibit a greater pore defect count due to their increased material volume, allowing more opportunities for gas to become entrapped. As shown in the lateral section, the FGMPS scaffold’s gradient design results in a gradual distribution of micro-pore defects across its structure, which is consistent with its varying strut diameters. Similar findings have been reported by Xiong et al. (2021), who investigated the effects of porosity gradient patterns on the mechanical performance of FGPS fabricated using laser powder bed fusion. Their study demonstrated that micro-pore defects were often concentrated near nodal areas, where structural complexity increases the likelihood of defect formation. Figure 5C illustrates the relationship between pore volume and pore sphericity for each scaffold type. Sphericity is used as an indicator of how closely the shape of a pore approaches that of a perfect sphere, with a value of 1 representing an ideal spherical shape (Sweijen et al., 2020). It is observed that smaller pores generally exhibit higher sphericity in all scaffold types. As the pore volume increases, the sphericity tends to decrease. However, it is important to note that the overall sphericity of these pores remains relatively high, indicating that they are still primarily gas-induced rather than being due to incomplete fusion. Unlike the irregular, low-sphericity pores typically associated with fusion defects, these spherical gas-induced micropores do not significantly compromise the structural integrity of the scaffold. This observation is consistent with findings reported in multiple studies, which have demonstrated that gas-induced micro-pore defects, due to their geometric regularity, do not substantially affect the mechanical strength of the samples (Li et al., 2024c; Spierings et al., 2011; Guo et al., 2022). Moreover, from a biocompatibility perspective, these micro-pores do not hinder cell adhesion or bone ingrowth, as they are predominantly located within the struts rather than on the scaffold surface.

### 3.4 Mechanical properties

**FIGURE 6:**
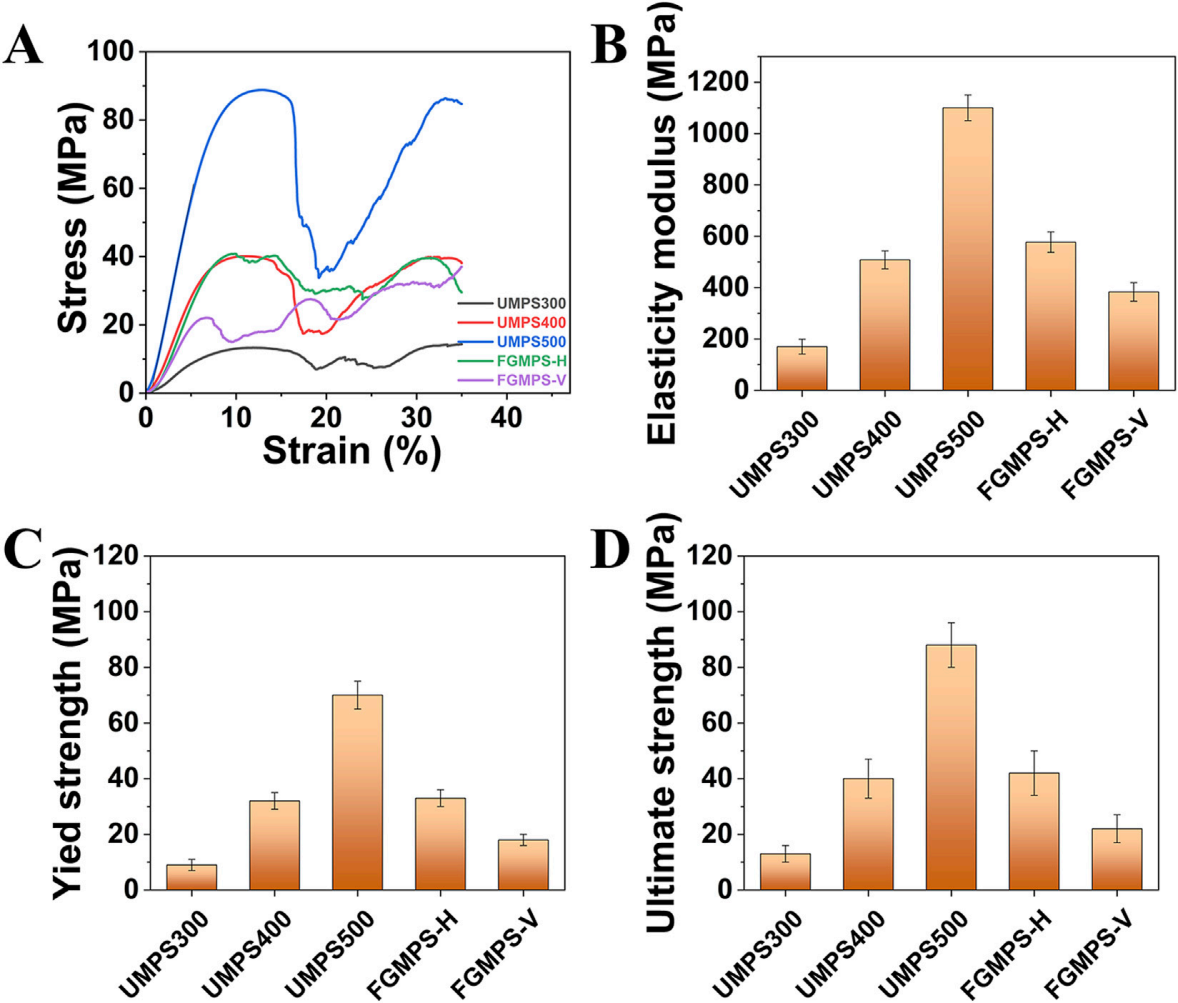
Mechanical properties of scaffolds under uniaxial compression. **(A)** Stress-strain curves for UMPS300, UMPS400, UMPS500, FGMPS-H, and FGMPS-V scaffolds. **(B–D)** Elastic modulus, yield strength, and ultimate strength of the scaffolds.

The mechanical properties of the porous scaffolds were assessed through uniaxial compression tests, focusing on elasticity modulus, yield strength, and ultimate strength, as depicted in Figure 6. The results provide insight into the influence of design variations on mechanical behavior. The typical stress-strain curves for the different scaffold types are shown in Figure 6A. The stress-strain curves for UMPS300, UMPS400, and UMPS500 show a similar pattern with three main stages: an initial linear elastic stage, where stress increases proportionally with strain; a yield stage, where stress reaches a peak and either stabilizes or decreases; and a fluctuation stage, where stress fluctuates as the scaffold undergoes deformation and redistribution. The FGMPS-H scaffold, oriented with the compression direction perpendicular to its gradient structure, exhibited two distinct stress peaks before overall structural failure. This may be attributed to that gradient appears to influence its load-bearing behavior by serving as a structural barrier during compression. As the gradient porous layers engage sequentially, they effectively impede the progression of localized failure, thereby extending the overall deformation process. This staggered engagement of the gradient structure likely mitigates the rapid collapse of weaker regions, instead distributing the strain across the scaffold. In contract, a markedly different response is observed for FGMPS-V, where the compression axis is aligned with the gradient direction. This configuration means that during compression, the layers with varying strut diameters are progressively engaged in the load-bearing process. Initially, the layers with smaller strut diameters undergo early collapse due to their lower load-bearing capacity. As compression continues, the regions with increasing strut diameters engage, resulting in the emergence of new stress peaks. This sequential collapse and subsequent strengthening of FGMPS-V are aligned with the findings of Zhang X-Y. et al., (2018), who demonstrated that a gradient porous scaffold with increasing density along the compression direction leads to progressive collapse of weaker layers followed by engagement of stiffer ones, enhancing both mechanical stability and energy absorption during deformation. Similarly, Chen et al. reported that the vertical gradient (VG) porous structures exhibited step-like stress responses, where weaker struts collapsed first, followed by a gradual increase in load-bearing by the remaining structure (Chen et al., 2023).

UMPS500 exhibited the highest elastic modulus (1,100 MPa), the highest yield strength (70 MPa), and the highest ultimate strength (88 MPa). This is primarily attributed to its higher structural density and reduced porosity, which contribute to greater load-bearing capacity. These values are substantially higher than the typical range for cancellous bone (50–500 MPa) (Henkel et al., 2013; Ghouse et al., 2019), indicating that UMPS500 is suited for regions requiring significant support. The remaining UMPS and FGMPS scaffolds exhibit properties within or slightly above the range for cancellous bone. In detail, the FGMPS-V presented an average elastic modulus of 383 MPa and a lower yield strength (22 MPa). Conversely, the FGMPS-H shows an average elastic modulus of 577 MPa and a strength of 40 MPa. This suggests they are appropriate for cavity bone defect reconstruction, offering moderate stiffness and strength to facilitate effective load transfer while avoiding excessive rigidity. Additionally, the graded porosity of the FGMPS scaffold enables more efficient load distribution, allowing different regions to progressively share mechanical loads and reducing stress concentration, which helps minimize stress shielding and enhances bone integration.

### 3.5 Clinical application prospects

**FIGURE 7:**
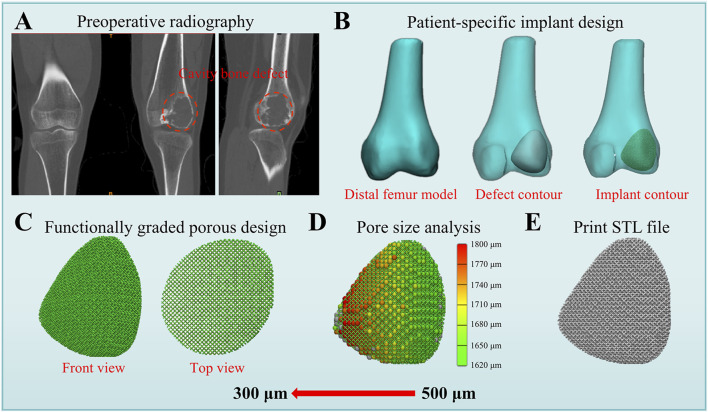
Workflow for patient-specific implant design and functional graded porous structure. **(A)** Preoperative radiography showing the distal femur with cavity bone defect. **(B)** Patient-specific implant design process, including the creation of the distal femur model, defect contour, and implant contour. **(C)** Functionally graded porous structure design displayed in front and top views, demonstrating a gradient pore distribution. **(D)** Pore size analysis of this patient-specific implant. **(E)** Final STL file generated for 3D printing.

The FGMPS holds immense potential for clinical application in the reconstruction of cavitary bone defects. One of the primary advantages of FGMPS lies in its ability to mimic the biomechanical properties of metaphyseal bone. Traditional implants often fail to balance load distribution adequately, leading to stress shielding or, conversely, inadequate mechanical stability. Figure 7 shows the workflow for patient-specific implant design and functional graded porous structure. As shown in Figure 7B, the design of patient-specific implant involves creating a 3D model of the distal femur, identifying the defect contour, and designing an implant that fits precisely within the defect. This approach ensures that the implant geometry is well-suited to the individual’s anatomy. Figure 7C illustrates the FGMPS design, showing both the front and top views of the scaffold. This design allows for effective load transfer and mechanical adaptability, which is particularly important for metaphyseal defect reconstruction.

## 4 Conclusion

This study successfully developed a functionally graded macro-porous scaffold (FGMPS) using selective laser melting for cavitary bone defect reconstruction. The FGMPS mimicked the natural density gradient of metaphyseal bone. Micro-CT analysis demonstrated high fabrication accuracy and structural fidelity. Compression tests confirmed mechanical properties suitable for cancellous bone. These findings highlight the FGMPS as a promising, personalized solution for cavitary bone defect repair.

## Data Availability

The original contributions presented in the study are included in the article/Supplementary Material, further inquiries can be directed to the corresponding authors.

## References

[B1] Aufa A. Hassan M. Z. Ismail Z. (2022). Recent advances in Ti-6Al-4V additively manufactured by selective laser melting for biomedical implants: prospect development. J. Alloys Compd. 896, 163072. 10.1016/j.jallcom.2021.163072

[B2] Breish F. Hamm C. Andresen S. (2024). Nature’s load-bearing design principles and their application in engineering: a review. Biomimetics 9 (9), 545. 10.3390/biomimetics9090545 39329566 PMC11430629

[B3] Caiazzo F. Alfieri V. Campanelli S. L. Errico V. (2022). Additive manufacturing and mechanical testing of functionally-graded steel strut-based lattice structures. J. Manuf. Process. 83, 717–28. 10.1016/j.jmapro.2022.09.031

[B4] Chao L. He Y. Gu J. Xie D. Yang Y. Shen L. (2023). Design of porous structure based on the Voronoi diagram and stress line for better stress shielding relief and permeability. J. Mater. Res. Technol. 25, 1719–34. 10.1016/j.jmrt.2023.05.282

[B5] Chen H. Han Q. Wang C. Liu Y. Chen B. Wang J. (2020). Porous scaffold design for additive manufacturing in orthopedics: a review. Front. Bioeng. Biotechnol. 8, 609. 10.3389/fbioe.2020.00609 32626698 PMC7311579

[B6] Chen J. Fan M. Zhou L. Chen W. Ren Y. Li W. (2023). The effect of microstructure on the fatigue behavior of titanium alloy graded porous structures fabricated by selective laser melting. J. Mater. Res. Technol. 27, 4290–304. 10.1016/j.jmrt.2023.10.281

[B7] Chung S. King M. W. (2011). Design concepts and strategies for tissue engineering scaffolds. Biotechnol. Appl. Biochem. 58 (6), 423–38. 10.1002/bab.60 22172105

[B8] Currey J. D. (2012). The structure and mechanics of bone. J. Mater. Sci. 47, 41–54. 10.1007/s10853-011-5914-9

[B9] Dalfino S. Savadori P. Piazzoni M. Connelly S. T. Giannì A. B. Del Fabbro M. (2023). Regeneration of critical‐sized mandibular defects using 3D‐printed composite scaffolds: a quantitative evaluation of new bone formation in *in vivo* studies. Adv. Healthc. Mater. 12 (21), 2300128. 10.1002/adhm.202300128 37186456 PMC11469182

[B10] Debroy T. Wei H. L. Zuback J. S. Mukherjee T. Elmer J. Milewski J. (2018). Additive manufacturing of metallic components–process, structure and properties. Prog. Mater. Sci. 92, 112–224. 10.1016/j.pmatsci.2017.10.001

[B11] Gao X. Tao C. Wu S. Chen B. (2022). Influence of modified microstructures and characterized defects on tensile properties and anisotropy of selective laser melting-produced Ti6Al4V alloys. J. Mater. Eng. Perform. 31 (9), 7705–18. 10.1007/s11665-022-06745-0

[B12] Georgeanu V. A. Gingu O. Antoniac I. V. Manolea H. O. (2023). Current options and future perspectives on bone graft and biomaterials substitutes for bone repair, from clinical needs to advanced biomaterials research. Appl. Sci. 13 (14), 8471. 10.3390/app13148471

[B13] Ghouse S. Reznikov N. Boughton O. R. Babu S. Ng K. G. Blunn G. (2019). The design and *in vivo* testing of a locally stiffness-matched porous scaffold. Appl. Mater. today 15, 377–88. 10.1016/j.apmt.2019.02.017 31281871 PMC6609455

[B14] Guo W. Feng B. Yang Y. Ren Y. Liu Y. Yang H. (2022). Effect of laser scanning speed on the microstructure, phase transformation and mechanical property of NiTi alloys fabricated by LPBF. Mater. and Des. 215, 110460. 10.1016/j.matdes.2022.110460

[B15] Henkel J. Woodruff M. A. Epari D. R. Steck R. Glatt V. Dickinson I. C. (2013). Bone regeneration based on tissue engineering conceptions—a 21st century perspective. Bone Res. 1 (1), 216–48. 10.4248/br201303002 26273505 PMC4472104

[B16] Hoque M. E. Showva N.-N. Ahmed M. Rashid A. B. Sadique S. E. El-Bialy T. (2022). RETRACTED: titanium and titanium alloys in dentistry: current trends, recent developments, and future prospects. Heliyon 8 (11), e11300. 10.1016/j.heliyon.2022.e11300 36387463 PMC9640965

[B17] Horstmann P. F. Hettwer W. H. Petersen M. M. (2018). Treatment of benign and borderline bone tumors with combined curettage and bone defect reconstruction. J. Orthop. Surg. 26 (3), 2309499018774929. 10.1177/2309499018774929 30428758

[B18] Hou G. Liu B. Tian Y. Liu Z. Zhou F. Ji H. (2020). An innovative strategy to treat large metaphyseal segmental femoral bone defect using customized design and 3D printed micro-porous prosthesis: a prospective clinical study. J. Mater. Sci. Mater. Med. 31, 66–9. 10.1007/s10856-020-06406-5 32696168

[B19] Huang E. E. Zhang N. Shen H. Li X. Maruyama M. Utsunomiya T. (2022). Novel techniques and future perspective for investigating critical-size bone defects. Bioengineering 9 (4), 171. 10.3390/bioengineering9040171 35447731 PMC9027954

[B20] Kaur M. Singh K. (2019). Review on titanium and titanium based alloys as biomaterials for orthopaedic applications. Mater. Sci. Eng. C 102, 844–62. 10.1016/j.msec.2019.04.064 31147056

[B21] Li J. Wu H. Liu H. Zuo D. (2023). Surface and property characterization of selective laser-melted Ti-6Al-4V alloy after laser polishing. Int. J. Adv. Manuf. Technol. 128 (1-2), 703–14. 10.1007/s00170-023-11880-6

[B22] Li Z. Lu M. Lei H. Zou S. Liu R. Wang Y. (2024c). Selective laser melting fabrication of body-temperature shape memory NiTi alloy for 4D-printed orthopedic implants. Virtual Phys. Prototyp. 19 (1), e2413181. 10.1080/17452759.2024.2413181

[B23] Li Z. Lu M. Zhang Y. Wang J. Wang Y. Gong T. (2024a). Intercalary prosthetic reconstruction with three‐dimensional‐printed custom‐made porous component for defects of long bones with short residual bone segments after tumor resection. Orthop. Surg. 16 (2), 374–82. 10.1111/os.13969 38111053 PMC10834207

[B24] Li Z. Lu M. Zhang Y. Wang J. Wang Y. Gong T. (2024b). 3D‐Printed personalized lattice implant as an innovative strategy to reconstruct geographic defects in load‐bearing bones. Orthop. Surg. 16 (4), 821–9. 10.1111/os.14003 38296795 PMC10984818

[B25] López A. Mestres G. Ott M. K. Engqvist H. Ferguson S. J. Persson C. (2014). Compressive mechanical properties and cytocompatibility of bone-compliant, linoleic acid-modified bone cement in a bovine model. J. Mech. Behav. Biomed. Mater. 32, 245–56. 10.1016/j.jmbbm.2014.01.002 24508711

[B26] Lv Y. Wang B. Liu G. Tang Y. Lu E. Xie K. (2021). Metal material, properties and design methods of porous biomedical scaffolds for additive manufacturing: a review. Front. Bioeng. Biotechnol. 9, 641130. 10.3389/fbioe.2021.641130 33842445 PMC8033174

[B27] Mauffrey C. Barlow B. T. Smith W. (2015). Management of segmental bone defects. JAAOS-Journal Am. Acad. Orthop. Surg. 23 (3), 143–53. 10.5435/jaaos-d-14-00018r1 25716002

[B28] Melancon D. Bagheri Z. Johnston R. Liu L. Tanzer M. Pasini D. (2017). Mechanical characterization of structurally porous biomaterials built via additive manufacturing: experiments, predictive models, and design maps for load-bearing bone replacement implants. Acta biomater. 63, 350–68. 10.1016/j.actbio.2017.09.013 28927929

[B29] Murr L. E. Gaytan S. M. Ramirez D. A. Martinez E. Hernandez J. Amato K. N. (2012). Metal fabrication by additive manufacturing using laser and electron beam melting technologies. J. Mater. Sci. and Technol. 28 (1), 1–14. 10.1016/s1005-0302(12)60016-4

[B30] Naghavi S. A. Tamaddon M. Garcia-Souto P. Moazen M. Taylor S. Hua J. (2023). A novel hybrid design and modelling of a customised graded Ti-6Al-4V porous hip implant to reduce stress-shielding: an experimental and numerical analysis. Front. Bioeng. Biotechnol. 11, 1092361. 10.3389/fbioe.2023.1092361 36777247 PMC9910359

[B31] Robinson P. G. Abrams G. D. Sherman S. L. Safran M. R. Murray I. R. (2020). Autologous bone grafting. Operative Tech. Sports Med. 28 (4), 150780. 10.1016/j.otsm.2020.150780

[B32] Spierings A. B. Schneider M. U. Eggenberger R. (2011). Comparison of density measurement techniques for additive manufactured metallic parts. Rapid Prototyp. J. 17 (5), 380–6. 10.1108/13552541111156504

[B33] Spierings A. B. Starr T. L. Wegener K. (2013). Fatigue performance of additive manufactured metallic parts. Rapid Prototyp. J. 19 (2), 88–94. 10.1108/13552541311302932

[B34] Sweijen T. Hassanizadeh S. M. Aslannejad H. Leszczynski S. (2020). The effect of particle shape on porosity of swelling granular materials: discrete element method and the multi-sphere approximation. Powder Technol. 360, 1295–304. 10.1016/j.powtec.2019.09.036

[B35] Tarchala M. Harvey E. J. Barralet J. (2016). Biomaterial‐stabilized soft tissue healing for healing of critical‐sized bone defects: the Masquelet technique. Adv. Healthc. Mater. 5 (6), 630–40. 10.1002/adhm.201500793 26855349

[B36] Trevisan F. Calignano F. Aversa A. Marchese G. Lombardi M. Biamino S. (2018). Additive manufacturing of titanium alloys in the biomedical field: processes, properties and applications. J. Appl. biomaterials and Funct. Mater. 16 (2), 57–67. 10.5301/jabfm.5000371 28967051

[B37] Wang W. Yeung K. W. (2017). Bone grafts and biomaterials substitutes for bone defect repair: a review. Bioact. Mater. 2 (4), 224–47. 10.1016/j.bioactmat.2017.05.007 29744432 PMC5935655

[B38] Wang X. Xu S. Zhou S. Xu W. Leary M. Choong P. (2016). Topological design and additive manufacturing of porous metals for bone scaffolds and orthopaedic implants: a review. Biomaterials 83, 127–41. 10.1016/j.biomaterials.2016.01.012 26773669

[B39] Xiong Y. Han Z. Qin J. Dong L. Zhang H. Wang Y. (2021). Effects of porosity gradient pattern on mechanical performance of additive manufactured Ti-6Al-4V functionally graded porous structure. Mater. and Des. 208, 109911. 10.1016/j.matdes.2021.109911

[B40] Yap C. Y. Chua C. K. Dong Z. L. Liu Z. H. Zhang D. Q. Loh L. E. (2015). Review of selective laser melting: materials and applications. Appl. Phys. Rev. 2 (4). 10.1063/1.4935926

[B41] Yeganeh M. Shahryari Z. Talib Khanjar A. Hajizadeh Z. Shabani F. (2023). Inclusions and segregations in the selective Laser-melted alloys: a review. Coatings 13 (7), 1295. 10.3390/coatings13071295

[B42] Zhang X.-Y. Fang G. Xing L.-L. Liu W. Zhou J. (2018b). Effect of porosity variation strategy on the performance of functionally graded Ti-6Al-4V scaffolds for bone tissue engineering. Mater. and Des. 157, 523–38. 10.1016/j.matdes.2018.07.064

[B43] Zhang M. Matinlinna J. P. Tsoi J. K. Liu W. Cui X. Lu W. W. (2020). Recent developments in biomaterials for long-bone segmental defect reconstruction: a narrative overview. J. Orthop. Transl. 22, 26–33. 10.1016/j.jot.2019.09.005 PMC7231954 32440496

[B44] Zhang X. Leary M. Tang H. Song T. Qian M. (2018a). Selective electron beam manufactured Ti-6Al-4V lattice structures for orthopedic implant applications: current status and outstanding challenges. Curr. Opin. Solid State Mater. Sci. 22 (3), 75–99. 10.1016/j.cossms.2018.05.002

[B45] Zhang Y. Lu M. Min L. Wang J. Wang Y. Luo Y. (2021). Three-dimensional-printed porous implant combined with autograft reconstruction for giant cell tumor in proximal tibia. J. Orthop. Surg. Res. 16 (1), 286. 10.1186/s13018-021-02446-x 33926481 PMC8082833

