# Supplementary material for: Selective laser melting fabrication of functionally graded macro-porous Ti-6Al-4V scaffold for cavity bone defect reconstruction
Source: Front Bioeng Biotechnol. 2025 Apr 28;13:1550309. doi: 10.3389/fbioe.2025.1550309 (PMC12066661; doi:10.3389/fbioe.2025.1550309)
Supplement: Supplementary file 1 [file DataSheet1.docx]

**Supplementary**

1. **The pore size was assessed using the “pore sphere” method within the unit cell of the lattice structure.**

**
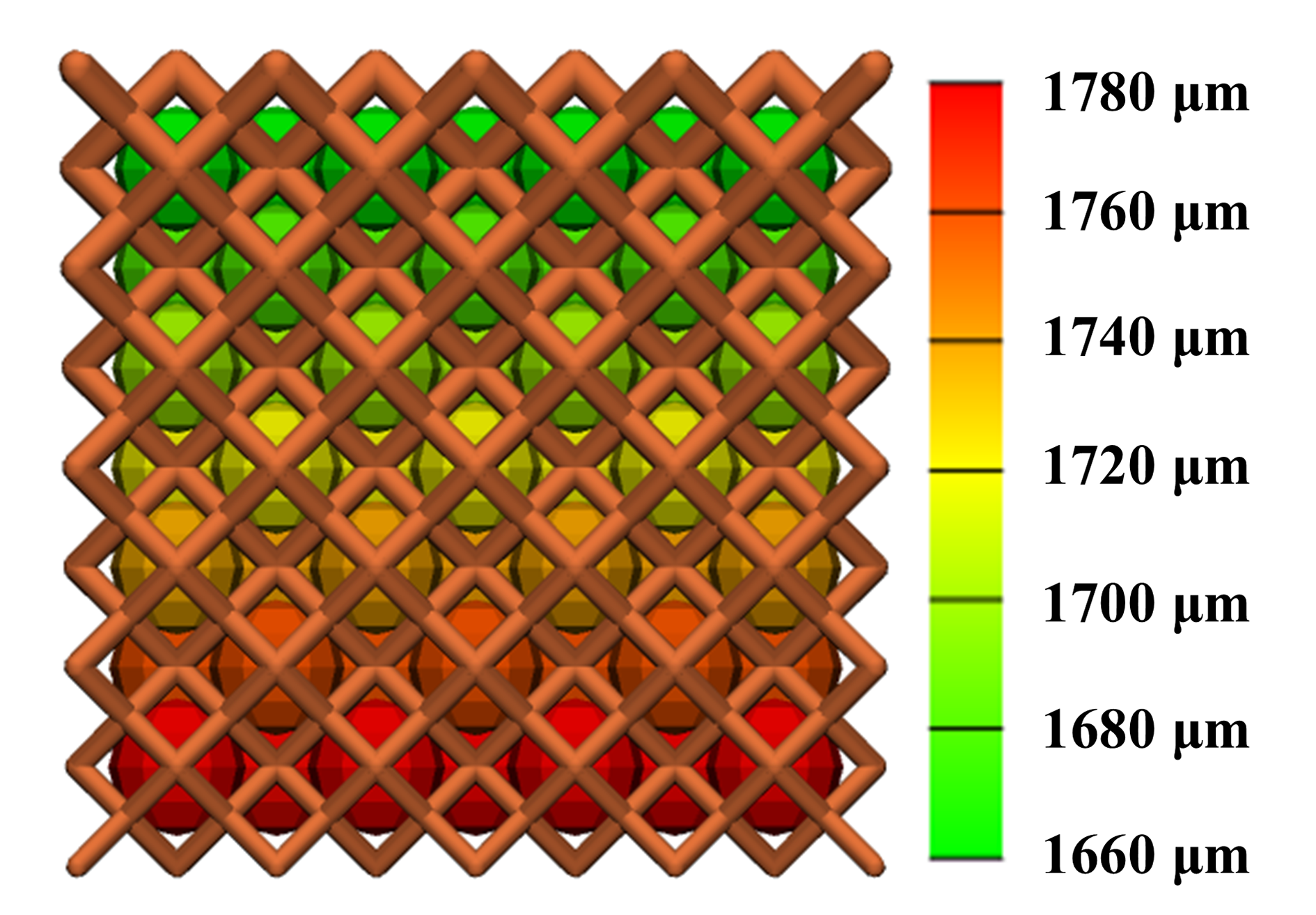
**
